# Supplementary material for: Polygenic adaptation to overnutrition reveals a role for cholinergic signaling in longevity
Source: bioRxiv. 2023 Jun 14:2023.06.14.544888. Preprint. [Version 1] doi: 10.1101/2023.06.14.544888 (PMC10312690; doi:10.1101/2023.06.14.544888)
Supplement: Supplement 1 [file NIHPP2023.06.14.544888v1-supplement-1.pdf]

bioRxiv preprint doi: https://doi.org/10.1101/2023.06.14.544888; this version posted June 14, 2023. The copyright holder for this preprint (which was not certified by peer review) is the author/funder, who has granted bioRxiv a license to display the preprint in perpetuity. It is made available under aCC-BY 4.0 International license.

Supplementary Figures

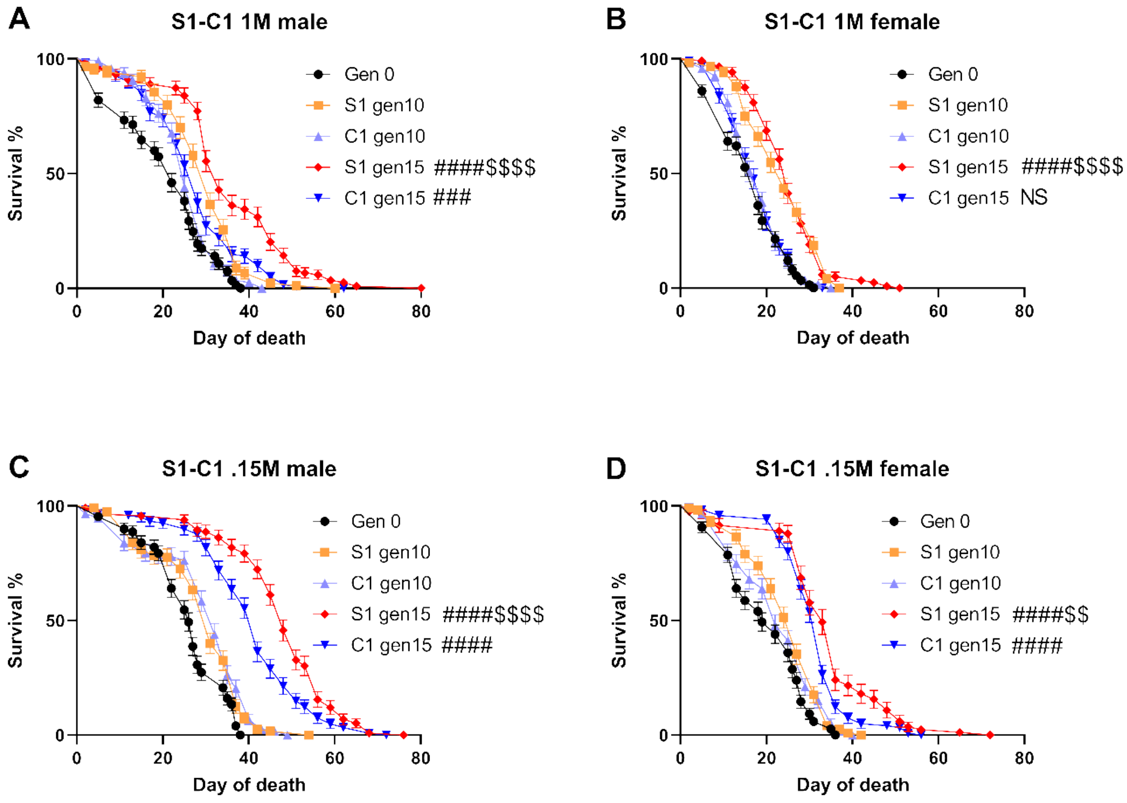

S1 Fig. Population survival of S1-C1 pair with confidence intervals.
In cohorts of 30, 120 recently eclosed mated males (A, C) from or females (B, D) were aged on 1M or 0.15M sucrose diets. Kaplan-Meier estimator curves for Generation 0 in black, Control populations are shown in cool colors, Selected populations in warm colors. The significance corresponding to number of symbols is as follows: *P < 0.05, **P < 0.01, ***P < 0.001, ****P < 0.0001 by log-rank Mantel-Cox. # represents the significance for generation 15 vs generation 0. $ represents the significance for the Selected population vs Control population at generation 15.

bioRxiv preprint doi: https://doi.org/10.1101/2023.06.14.544888; this version posted June 14, 2023. The copyright holder for this preprint (which was not certified by peer review) is the author/funder, who has granted bioRxiv a license to display the preprint in perpetuity. It is made available under aCC-BY 4.0 International license.

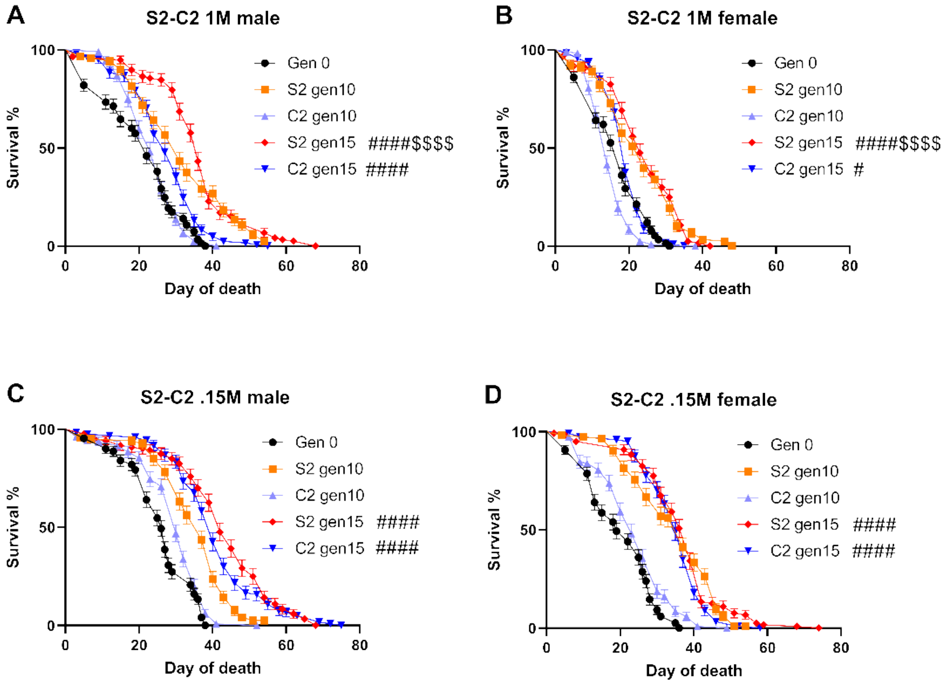

<u>S2 Fig. Population survival of S2-C2 pair with confidence intervals.</u>

In cohorts of 30, 120 recently eclosed mated males (A, C) from or females (B, D) were aged on 1M or 0.15M sucrose diets. Kaplan-Meier estimator curves for Generation 0 in black, Control populations are shown in cool colors, Selected populations in warm colors. The significance corresponding to number of symbols is as follows: *P < 0.05, **P < 0.01, ***P < 0.001, ****P < 0.0001 by log-rank Mantel-Cox. # represents the significance for generation 15 vs generation 0. $ represents the significance for the Selected population vs Control population at generation 15.

bioRxiv preprint doi: https://doi.org/10.1101/2023.06.14.544888; this version posted June 14, 2023. The copyright holder for this preprint (which was not certified by peer review) is the author/funder, who has granted bioRxiv a license to display the preprint in perpetuity. It is made available under aCC-BY 4.0 International license.

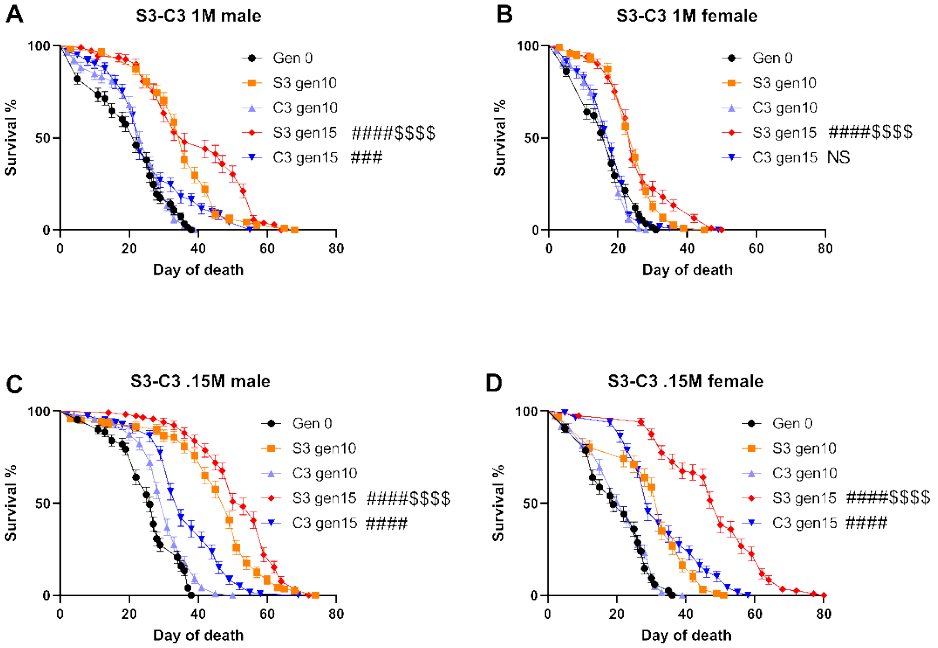

<u>S3 Fig. Population survival of S3-C3 pair with confidence intervals.</u>
In cohorts of 30, 120 recently eclosed mated males (A, C) from or females (B, D) were aged on 1M or 0.15M sucrose diets. Kaplan-Meier estimator curves for Generation 0 in black, Control populations are shown in cool colors, Selected populations in warm colors. The significance corresponding to number of symbols is as follows: *P < 0.05, **P < 0.01, ***P < 0.001, ****P < 0.0001 by log-rank Mantel-Cox. # represents the significance for generation 15 vs generation 0. $ represents the significance for the Selected population vs Control population at generation 15.

bioRxiv preprint doi: https://doi.org/10.1101/2023.06.14.544888; this version posted June 14, 2023. The copyright holder for this preprint (which was not certified by peer review) is the author/funder, who has granted bioRxiv a license to display the preprint in perpetuity. It is made available under aCC-BY 4.0 International license.

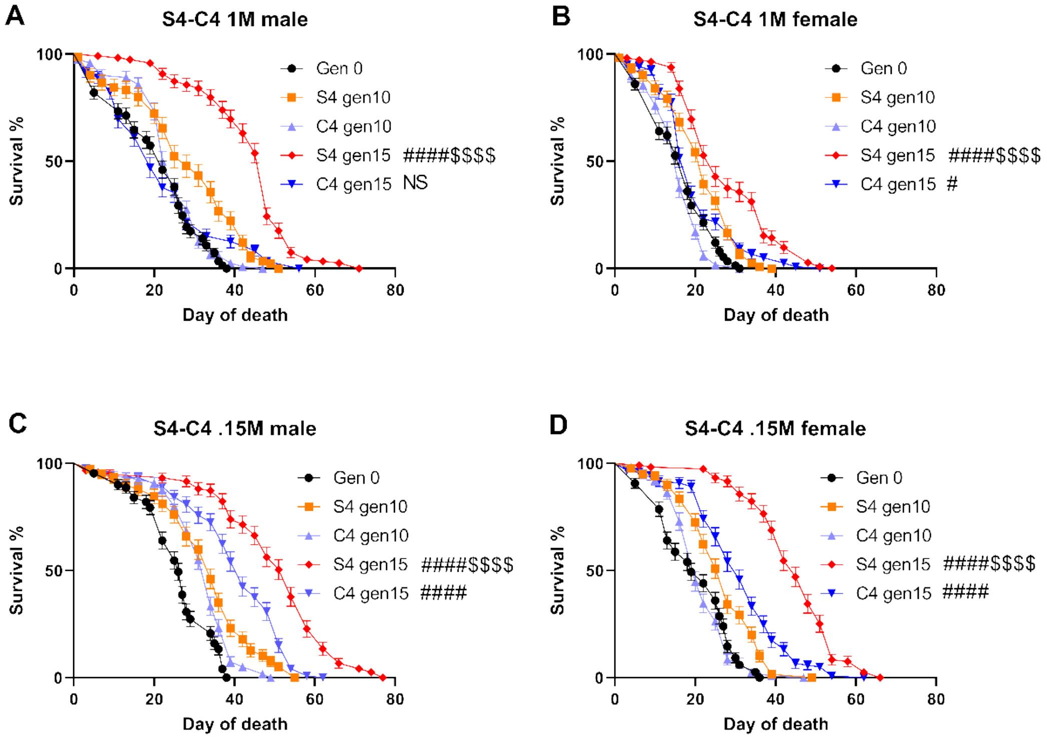

<u>S4 Fig. Population survival of S4-C4 pair with confidence intervals.</u>

In cohorts of 30, 120 recently eclosed mated males (A, C) from or females (B, D) were aged on 1M or 0.15M sucrose diets. Kaplan-Meier estimator curves for Generation 0 in black, Control populations are shown in cool colors, Selected populations in warm colors. The significance corresponding to number of symbols is as follows: *P < 0.05, **P < 0.01, ***P < 0.001, ****P < 0.0001 by log-rank Mantel-Cox. # represents the significance for generation 15 vs generation 0. $ represents the significance for the Selected population vs Control population at generation 15.

bioRxiv preprint doi: https://doi.org/10.1101/2023.06.14.544888; this version posted June 14, 2023. The copyright holder for this preprint (which was not certified by peer review) is the author/funder, who has granted bioRxiv a license to display the preprint in perpetuity. It is made available under aCC-BY 4.0 International license.

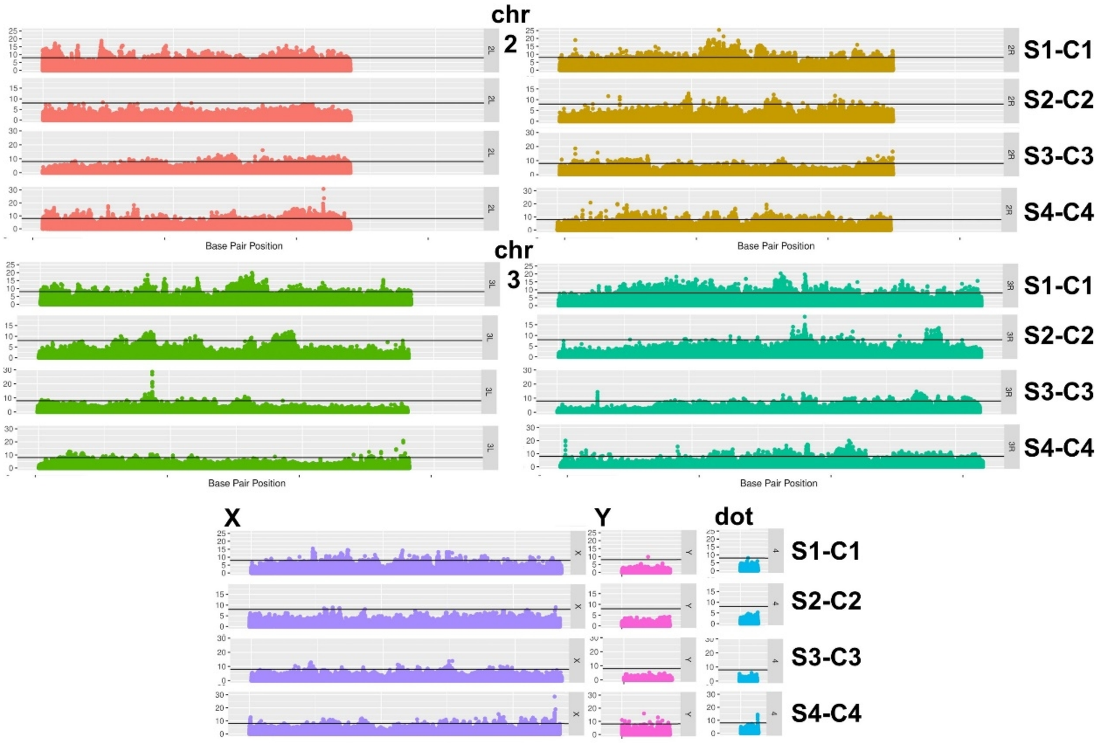

S5 Fig. Pooled whole-genome sequencing shows changes in allele frequencies after ten generations of selection. Manhattan plots show SNPs identified as significant deviations in allele frequencies between Selected populations and Control populations via a Fisher's exact test. Chromosomes are stacked for each of the different replicated comparisons. The horizontal line for each chromosome represents the P value cutoff (P < ~10^{-8}).

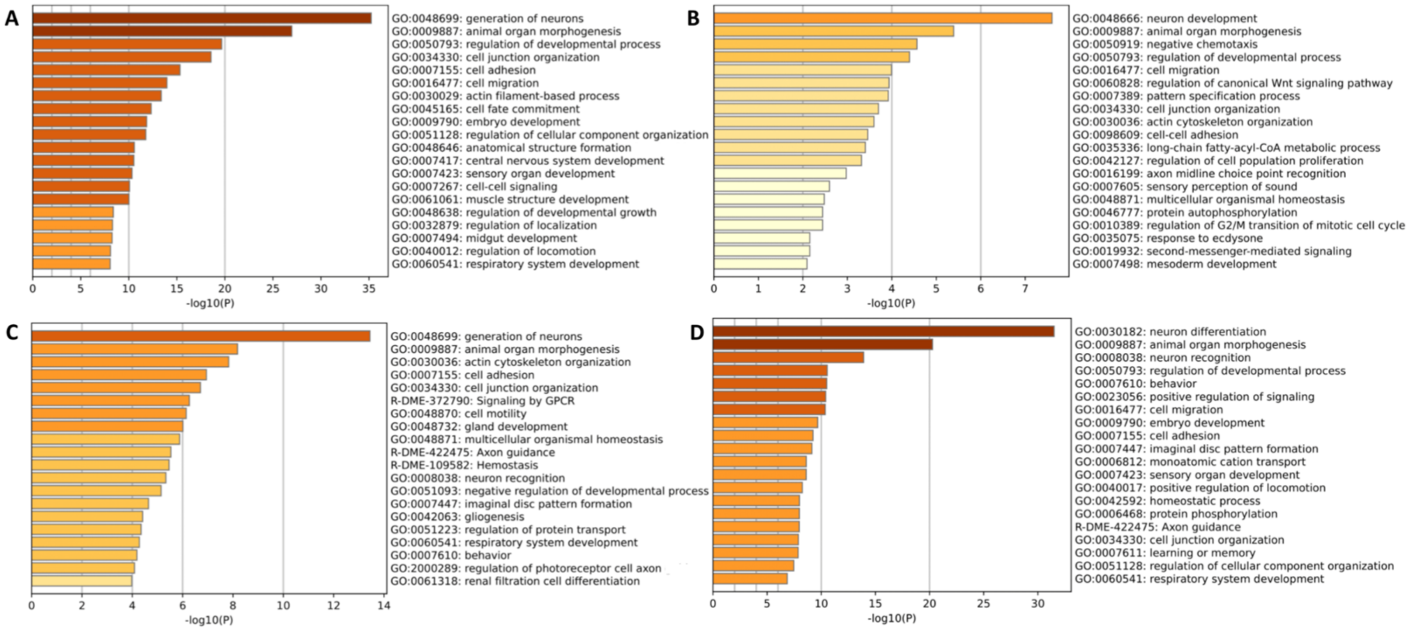

S6 Fig. Gene ontology enrichment analysis of population pairs. Figures show top 20 enriched ontological categories obtained from significantly differentiated loci (by Fisher's exact test) in (A) Populations S1-C1, (B) Populations S2-C2, (C) Populations S3-C3, and (D) S4-C4 population pairs.

bioRxiv preprint doi: https://doi.org/10.1101/2023.06.14.544888; this version posted June 14, 2023. The copyright holder for this preprint (which was not certified by peer review) is the author/funder, who has granted bioRxiv a license to display the preprint in perpetuity. It is made available under aCC-BY 4.0 International license.

Terms with P < 0.01 and enrichment score >1.5 were counted as significant categories (ref for Metascape: https://pubmed.ncbi.nlm.nih.gov/30944313/).

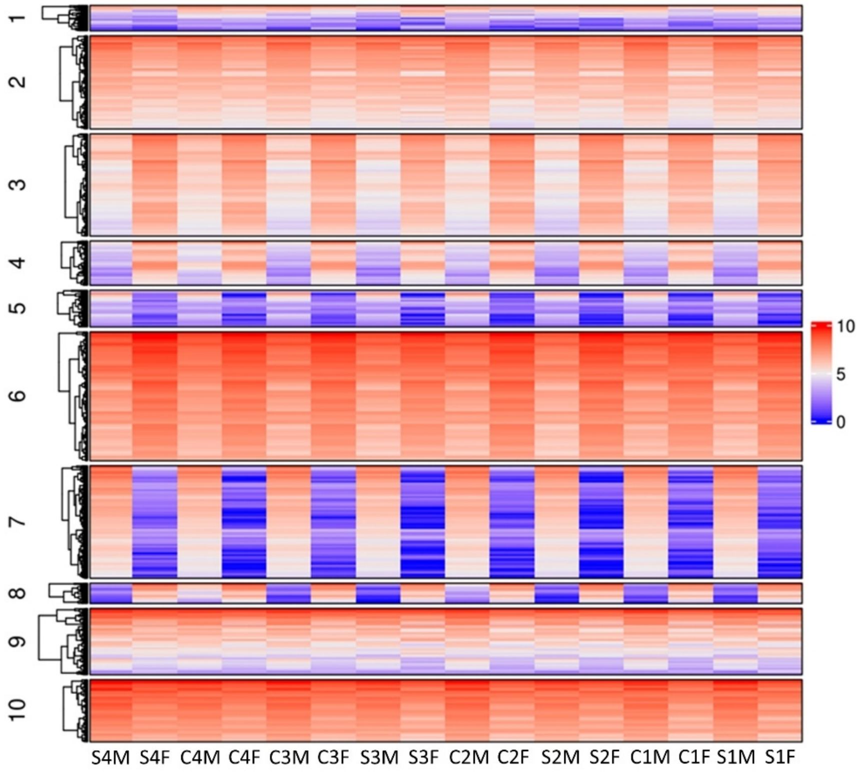

S7 Fig. Differential expression profiles in HS-selected flies.

Flies were fed HS for 1 week and whole animal RNA was used for RNA-seq. Cluster analysis of the median expression values from all populations for both males and females was conducted using a mixture of multivariate Poisson log-normal distributions. The models with number of clusters ranging from 1 to 15 were fitted and the model-selection criteria selected a model with 10 clusters. Log-transformed median expression profiles of all genes in all ten clusters are visualized using a heatmap.

bioRxiv preprint doi: https://doi.org/10.1101/2023.06.14.544888; this version posted June 14, 2023. The copyright holder for this preprint (which was not certified by peer review) is the author/funder, who has granted bioRxiv a license to display the preprint in perpetuity. It is made available under aCC-BY 4.0 International license.

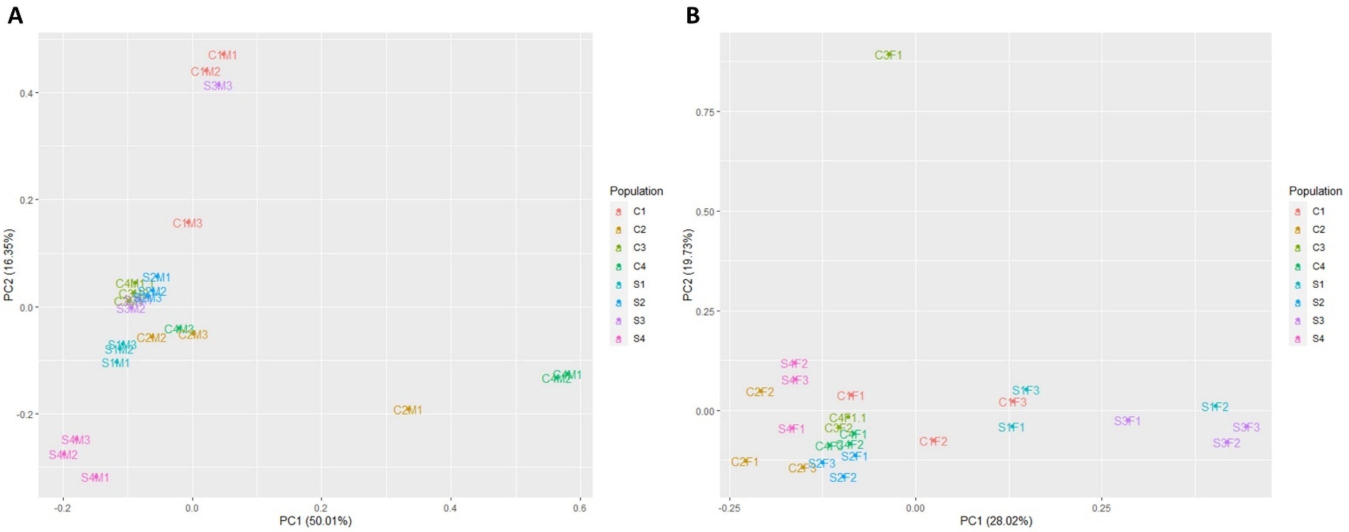

S8 Fig. Populations differentiate by gene expression at generation 10.
Adult male (A) and female (B) flies were fed HS for 1 week and whole animal RNA was used for RNA-seq. Principal component analysis (PCA) scores plots show the variance between normalized counts per million (CPM) of 3 replicates from control and selected populations. Colors represent populations.

Gene	Allele Frequency
mAChR-A	S1-0, S2-0, S3-0, S3-C4
mAChR-B	S1-0, S1-C1, S2-0, S3-0, S4-0
nAChRalpha1	S1-C1, S2-0, S3-0, S4-0
nAChRalpha3	S1-0, S1-C1, S2-0, S4-C4
nAChRalpha4	S1-0, S1-C1, S2-0
nAChRalpha5	S1-0, S1-C1, S2-0, S3-0, S3-C3, S4-0, S4-C4
nAChRalpha6	S1-0, S1-C1, S2-0, S3-0
nAChRalpha7	S2-0, S3-0
nAChRbeta1	S4-0
nAChRbeta2	S1-0, S1-C1, S2-0, S3-0, S4-0
nAChRbeta3	S1-0, S1-C1, S2-0
ChT	S1-0, S3-0

bioRxiv preprint doi: https://doi.org/10.1101/2023.06.14.544888; this version posted June 14, 2023. The copyright holder for this preprint (which was not certified by peer review) is the author/funder, who has granted bioRxiv a license to display the preprint in perpetuity. It is made available under aCC-BY 4.0 International license.

ChAT	S1-0, S2-0, S3-0, S4-0
VAChT	S1-0, S2-0, S2-C2, S4-0, S4-C4

S1 Table: Cholinergic genes with significant changes in HS-selected fly populations.
A diverse array of genes involved in cholinergic signaling (pre- and postsynaptic) were identified by both changes in expression and allele frequency in response to selection. Populations listed under allele frequency possess significant changes in allele frequency from either generation 0 (X-0) or from their paired control population at generation 10 (X-Y). ChT, choline transporter; ChAT, choline acetyltransferase; VAChT, vesicular acetylcholine transporter.

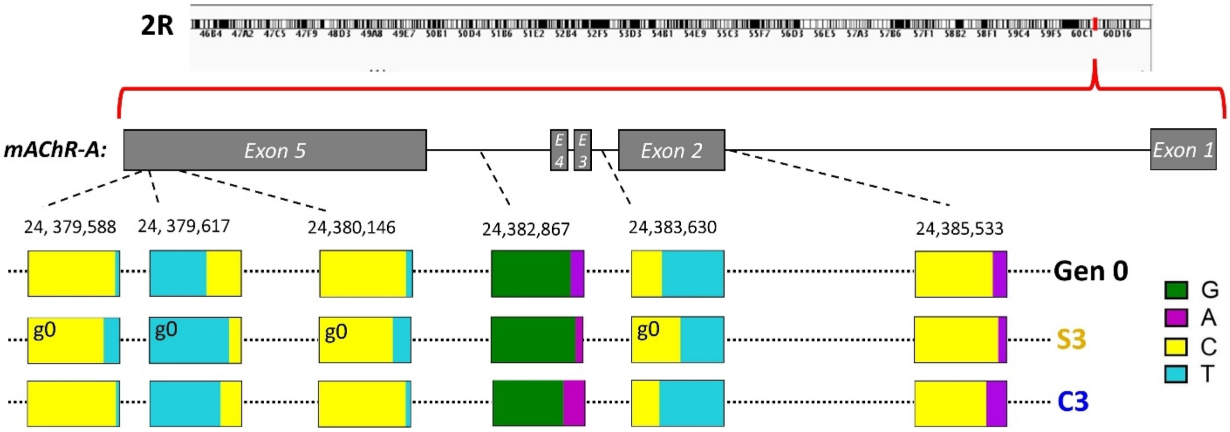

S9 Fig. Changes in allele frequency in *mAChR-A* observed in populations S3 and C3.
The top six changes ranked by lowest P value using a Fisher's exact test all differed with $P < 10^{-5}$ between S3 and C3. g0 denotes differences that reached the same degree of significance from generation 0. Generation 0, Selected population S3, and its corresponding Control population C3 are stacked to illustrate the major and minor allele frequencies.
